# Translation and Adaptation of the Online Gambling Disorder Questionnaire (OGD-Q) into Brazilian Portuguese

**DOI:** 10.1007/s10899-026-10480-9

**Published:** 2026-03-08

**Authors:** Maria Clara Siqueira Rego, Victor Hugo Maia de Souza, Leonardo Fernandes Martins, Breno Sanvicente-Vieira

**Affiliations:** Department of Psychology, Pontifical Catholic University of Rio de Janeiro, Rio de Janeiro, 2025 Brazil

**Keywords:** Gambling disorder, Online gambling, Psychometric properties, Assessment

## Abstract

Online gambling has grown increasingly popular over the past decade, especially since the COVID-19 pandemic. Online gamblers tend to face more issues than those who gamble in traditional settings, leading to a higher risk of developing pathological gambling. In Brazil, gambling activities are culturally accepted and normalized, which encourages widespread participation among the population. Therefore, screening and assessing online gambling is now a public necessity. The Online Gambling Disorder Questionnaire (OGD-Q), developed by González-Cabrera et al. (2020), aims to evaluate symptoms of gambling disorder primarily online. Given the significance of this issue in Brazil, this study sought to adapt the scale for cross-cultural use and examine its psychometric properties. The sample included 298 participants who completed an online survey. Nomothetic validity was tested through Spearman’s Rho correlations between measures of depression, anxiety, stress, and online gaming behavior. Internal consistency was assessed using Cronbach’s alpha and Omega Coefficient, along with confirmatory factor analysis. Results showed strong reliability, with a Cronbach’s alpha of 0.92 and an Omega Coefficient of 0.93. The confirmatory factor analysis supported the proposed one-factor structure, demonstrating good fit indices and factor loadings. Significant correlations were found in the total sample between OGD-Q scores and measures of online gaming, depression, anxiety, and stress. In summary, the findings demonstrate that the Brazilian version of OGD-Q is an effective tool for assessing online gambling disorder in the Brazilian population.

**Supplementary Information:**

The online version contains supplementary material available at 10.1007/s10899-026-10480-9.

## Introduction

A survey about gambling in the Brazilian population shows that 17,6% of Brazilians have gambled online in the past year, and 7,3% exhibit problematic gambling behaviors (Laranjeira et al., 2025). The internet and online activities influence society (Fussey & Roth, 2020) and also affect individuals, potentially leading to psychological harm (e.g., online sexual victimization, cyberbullying, and problematic internet use) (Machimbarrena et al., 2018). Additionally, even ancient psychological issues have evolved into new forms, such as gambling behavior (Gainsbury, 2015). Since internet commercialization, gambling is no longer confined to physical places like casinos and betting shops but is also accessible online (e.g., betting websites and online groups). Online gambling involves internet-based activities related to games and betting conducted remotely via tablets, computers, and smartphones (Gainsbury et al., 2015a, b). It is not separate from gambling but a specific form that can be done privately and without face-to-face interaction (Gainsbury et al., 2015a, b). Since COVID-19, online gambling activities have increased. The industry projects 290 million players by 2029 and market profits of $ 97.15 billion in 2024 (Statista Market Insights, (Statista 2024)). This change offers new perspectives for studying pathological gambling and gambling disorders.

Gambling disorder is marked by long-term problematic gambling behaviors that cause significant dysfunction or distress (American Psychiatric Association, 2022). The global prevalence of gambling disorder is estimated to be around 0.1% to 0.7% (American Psychiatric Association, 2022), but it can reach up to 6.5% depending on the country (Calado & Griffiths, 2016). Individuals with the disorder face a higher risk of developing psychological comorbidities (American Psychiatric Association, 2022; Dowling et al., 2015), most commonly: substance use disorders (7% to 17%) (Dowling et al., 2015; Håkansson et al., 2018), mood disorders (23% to 38%) (Dowling et al., 2015; Lorains et al., 2011), anxiety disorders (18% to 37%) (Lorains et al., 2011; Dowling et al., 2015), and personality disorders (10% to 78%) (Lorains et al., 2011).

It was estimated that between 1.4% and 2.8% of the population gambled each year in the early 90 s (Blume, 1995). Recent data show that 46.2% of the population gambles annually, and the prevalence of gambling disorder remains around 1.41% (Tran et al., 2024). The method of gambling is considered one of the main factors contributing to the observed changes (Ghelfi et al., 2023). This is especially relevant considering that a significant portion of gamblers today engage preferably in online betting: 32,1% in Brazil (Laranjeira et al., 2025), 22% in the United States (Siena College Research Institute, 2025), and 38% in the United Kingdom (Gambling Comission, 2024). In response to these changes, since 2019, the International Statistical Classification of Diseases and Related Health Problems (ICD-11) has included a specific category for Gambling disorder, called “Gambling disorder, predominantly online” (World Health Organization, 2019). Theories about gambling highlight ecological factors such as availability and increased access as important in developing the disorder (Abbott, 2020; Blaszczynski & Nower, 2002; Jacobs, 1986). Today, online gambling can be accessed anywhere at any time, which raises the risk of developing the disorder (Gainsbury et al., 2015; Gainsbury et al., 2015; González-Cabrera et al., 2020; Hing et al., 2014). Recent research shows that compared to offline gamblers, online gamblers tend to gamble more often, for longer durations, experience more gambling-related issues, spend more money, have a higher risk of developing pathological gambling, and typically begin gambling about 8.89 years earlier (Díaz & Pérez, 2021; Ghelfi et al., 2023; González-Cabrera et al., 2020).

## Gambling in Brazil

In Brazil, gambling regulation is not uniform across all activities. Law 14.790/2023 (Brazil, 2023) and Law 13.756/2018 (Brazil, 2018), respectively, legalize fixed-odds sports betting and official lotteries. Casinos, animal games (“jogo do bicho”), and slot machines are officially prohibited in Brazil under Law 3.688/1941 that mentions in the 50th article that holding or exploring gambling activities (wrote in Brazilian Portuguese in the document as “jogo de azar”, in literal English translation being “bad luck game” or “bad luck play”) is a fault configuring case for being arrested and fined (Brazil, 1941). However, such practices are classified as contraventions (i.e., illegal activities that are considered less serious than crimes).

Despite legal prohibitions and recent regulation of sports betting, gambling has long been embedded in Brazilian culture (Tavares, 2014). The cultural normalization of gambling dates back to the late 19th century, when the animal game (“jogo do bicho”) was introduced at Rio de Janeiro’s Zoo. Upon purchasing a zoo ticket, visitors received an image of one of 25 animals, and a random draw selected an animal, awarding prizes to ticket holders whose image matched that animal. Despite being illegal since 1941, the “animal game” has spread nationwide and remains a standard practice deeply rooted in Brazilian culture nowadays (Pena, 2024). Following the enduring popularity of the “animal game”, gambling in Brazil reappeared in socially accepted forms, such as the rise of casinos in the 1930s/40s, which became cultural and entertainment spaces until their prohibition in 1941 (Aguiar & Santos, 2022). Official and legal lotteries, later consolidated under Caixa Econômica Federal (a public financial institution), have consistently attracted millions of gamblers and remain the most popular gambling practice in Brazil (Laranjeira et al., 2025).

These historical continuities demonstrate how gambling has become normalized in Brazilian society. Even laws have renamed gambling practices: while Law 3.688/1941 initially referred to it as “jogo de azar” (“bad luck game”), more than 60 years later it was changed to “aposta” (“gamble”). In the latest Brazilian laws concerning gambling—14.790/2023 (Brazil, 2023) and 13.756/2018 (Brazil, 2018)—the original term is used only once, which highlights how the harm of gambling is being culturally concealed, and the behavior continues to grow and adapt to different formats and generations despite repeated restrictions.

Compared to American gamblers, Brazilian gamblers tend to progress more quickly from recreational to pathological gambling on electronic gambling machines, exhibiting behaviors such as continuing or increasing bets after losses in an attempt to recover the money. It highlights differences related to psychosocial factors. and cultural characteristics that impact gambling and require particular adaptations (Medeiros et al., 2015). Specifically, in Brazil, online gambling bets averaged 20% of the minimum wage in 2023, and half of bettors reported losing more than they won (Datafolha, 2023). Before the legalization of sports betting, a Brazilian gambler’s maximum monthly expenditure on gambling amounted to 5.4% of family income for social gamblers, 16.9% for problem gamblers, and 20% for pathological gamblers (Tavares et al., 2010). Recent data show that 25.9% of the Brazilian population has gambled at some point in their life, with 5.6% engaging in online gambling and 0.8% meeting the criteria for gambling disorder. Within this population, certain groups are identified as more vulnerable. Online gamblers have a significantly higher risk of developing gambling-related problems, with 66.8% of them classified as at risk, compared to 26.8% of gamblers involved in other activities. Although the law prohibits underage individuals from gambling on online betting platforms, 10% of teenagers reported having gambled in the year before data collection, and 55.2% of these were classified as at risk or exhibiting problematic gambling behaviors. Socioeconomically disadvantaged individuals, earning a minimum wage or less, show a higher prevalence of risky and problematic gambling behaviors, at a rate of 52.8%. Overall, 7.3% of the population exhibits problematic gambling behaviors (Laranjeira et al., 2025).

The cultural acceptance of gambling, along with the regulation of sports betting and the widespread advertising of gambling platforms among Brazilians, has increased access to gambling activities (Laranjeira et al., 2025). This has contributed to a mass phenomenon that causes social harm related to gambling, impacting not only individuals but also public health, as seen in the rising rates of gambling disorder and risky or problematic gambling behaviors, which demand societal responses to manage the outcomes (Kim et al., 2024; Tavares, 2014; Laranjeira et al., 2025).

### Online Gambling Assessment

Online gambling differs from land-based gambling in its structural features, including increased accessibility (available 24/7), a wider range of products, faster payment methods, higher internet speeds, anonymity, and extensive media advertising (Hing et al., 2022). These factors also influence gambler behavior, making participation easier and increasing the risk of harm (Ghelfi et al., 2023; Hing et al., 2022; Shaw & Williams, 2023). Compared to only offline gamblers, those who gamble exclusively online tend to engage more frequently, spend more time and money, and exhibit higher levels of problematic gambling behaviors (Shaw & Williams, 2023). As a result, there was a need to assess online gambling disorder symptoms. In response, González-Cabrera et al. (2020) developed the Online Gambling Disorder Questionnaire (OGD-Q), the first tool specifically designed to assess online gambling disorder. Its development aligns with the updated criteria for gambling disorder in the latest DSM-5 (American Psychiatric Association, 2022) and the ICD-11 (World Health Organization, 2019), which includes the classification for gambling disorder (6C50) and specifically “predominant online gambling” (6C50.1).

There were earlier scales assessing online gambling disorder, such as the Problem Gambling Severity Index (PGSI; Ferris & Wynne, 2001) and the South Oaks Gambling Screen (SOGS; Lesieur & Blume, 1987). The SOGS was based on the outdated DSM-III criteria for gambling disorder (Lesieur & Blume, 1987), just adding online adaptation to items. Similarly, the PGSI measures rates of problem gambling based on the outdated DSM-IV, with its other items focusing on indicators of gambling involvement (Ferris & Wynne, 2001). Neither instrument was developed in accordance with contemporary psychometric standards, as with the OGD-Q. The PGSI reported internal consistency of α = 0.84 in its original development study, based on exploratory factor analysis supporting a single-factor structure; however, no omega coefficients, item-level factor loadings, or convergent validity analyses were presented (Ferris & Wynne, 2001). Similarly, the original SOGS validation showed acceptable internal consistency (α ≈ 0.97 in clinical samples and lower values in community samples) (Lesieur & Blume, 1987). However, no confirmatory factor analysis or model-based construct validation was conducted, and psychometric evidence was limited to item counts rather than to latent structure. Thus, PGSI and SOGS are influential and relevant historical measures for gambling assessment. However, both instruments lack modern psychometric evaluation and a theoretical background specifically for online gambling. In contrast, OGD-Q was designed and tested using contemporary psychometric criteria, providing more substantial evidence for construct validity and item functioning.

## The Online Gambling Disorder Questionnaire (OGD-Q)

As mentioned, OGD-Q (González-Cabrera et al., 2020) was designed to identify online gambling disorder based on updated manuals and expert recommendations. The questionnaire consists of 11 items and employs a 5-point scale, ranging from 1 (never) to 5 (every day), with higher scores indicating greater involvement in online gambling. The OGD-Q was initially tested and validated in a cross-sectional study involving 2,691 students from 16 schools across seven Spanish regions. The final sample included 883 students with online gambling experience, most of whom were boys. An exploratory factor analysis (EFA) was conducted, revealing a single factor that accounted for 68.6% of the total variance. A confirmatory factor analysis (CFA) on a one-factor model indicated a good fit with the following indices: χ2 (44) = 87.523, *p* < 0.001; RMSEA = 0.010 (95% CI [0.000, 0.013]); CFI = 0.998; NNFI = 0.997; and SRMR = 0.037. In the original study, the OGD-Q showed Cronbach’s alpha and omega coefficients of 0.94 and 0.95, respectively (González-Cabrera et al., 2020), which are higher than those of the PGSI (Ferris & Wynne, 2001) and comparable to those reported for the SOGS in clinical samples (Lesieur & Blume, 1987), but higher than those from community members. Later, the instrument’s psychometric properties were evaluated in a sample of 968 online gamblers aged 18 to 64 years (Stavropoulos et al., 2022). In this study, the Cronbach’s alpha and McDonald’s omega were both 0.95 (Stavropoulos et al., 2022).

## The Present Study

This study aimed to translate and adapt the OGD-Q into Brazilian Portuguese and to evaluate its psychometric properties in a Brazilian sample. This initiative is an important effort to address the growing impact of gambling in Brazil. As mentioned, gambling is increasingly becoming more popular, particularly through online betting, resulting in substantial social and health problems (Ghelfi et al., 2023; Shaw & Williams, 2023). The recognition of online gambling patterns as a specific and distinct form of gambling disorder in ICD-11 (World Health Organization, 2019) highlights the importance of developing a valid measure, particularly considering Brazil’s status as one of the world’s most populous countries. Since gambling is becoming a public health concern and a crisis in the country (Laranjeira et al., 2025), it is urgent to provide tools that can easily and accurately assess online gambling disorder. The OGD-Q was specifically chosen because it was developed based on the latest manuals (González-Cabrera et al., 2020), recommendations on online gambling, and psychometric principles. Lastly, testing its psychometric properties among Brazilians is necessary, as cultural differences can influence gambling behavior (Medeiros et al., 2015).

## Methods

### Design and Participants

This was a cross-sectional online study. The sample was drawn from the general population. To be included, all participants had to identify as Brazilian, reside in Brazil, be able to read and write in Brazilian Portuguese, and have gambled online at least once in the past 12 months. Participants who began the protocol but did not complete it were excluded. The initial sample comprised 393 participants. Participants who did not respond to all questionnaires were excluded (*n* = 95). The final sample comprised 298 participants.

The rationale for sample size was based on recommendations by Mundfrom et al. (2005) for factor analyses. Although no absolute minimum sample size is provided, the authors suggest a guideline of 3–20 individuals per variable. For our one‑factor instrument with 11 tested items, this corresponds to a recommended range of 33–220 participants (Mundfrom et al., 2005). In addition, Kline (2016) recommends a minimum of approximately 200 participants for confirmatory factor analysis to ensure stable parameter estimates. Because of missing data in online surveys, we considered including at least 35% more responses than the maximum recommended number of participants (i.e., 220), which means a minimum estimation of 297 participants, exceeding the thresholds proposed by Mundfrom et al. (2005) and the benchmark suggested by Kline (2016), supporting the adequacy of the CFA. In line with this, we achieved 298 valid participants.

### Instruments

All instruments were included in an online survey—a sociodemographic questionnaire collected data on age, education, ethnicity, gender, and physical disability status.

The Online Gambling Disorder Questionnaire (OGD-Q) (González-Cabrera et al., 2020) is a general measure of online gambling and related problematic symptoms. Although its initial validation study was conducted with adolescents, the scale was developed to capture core features of online gambling disorder at any age. This 11-item questionnaire uses a 5-point scale, from one (1) as “never” to five (5) as “every day”. A higher score on the assessment scale indicates a greater intensity of online gambling behavior, ranging from 11 to 55. The Cronbach’s alpha and omega coefficients for this survey were 0.94 and 0.95, respectively. Any item with a score of 3 or higher, “often” (3), “very often” (4), and “every day” (5), was classified as a “problem”, and items scoring below 3, “never” (1) and “occasionally” (2), were classified as “no problem”. Criteria for a diagnosis of “online gambling disorder” were met when individuals scored four (4) or higher on four (4) items or more in the past 12 months. Participants who met this criterion for six to 12 months were classified as having an “online gambling problem”. Participants with four or more indicators for less than six months were classified as “at risk for online gambling problems”. We conducted a translation and adaptation of the OGD-Q in this work; the procedures are described in the following.

The Internet Gaming Disorder Scale – Short Form (IGDS9-SF) (Donadon et al., 2020; Pontes & Griffiths, 2015) evaluated the severity of IGD symptoms and the consequences of excessive online gaming behavior. This one-dimensional tool includes nine items based on the DSM-5 criteria for IGD. Participants respond on a 5-point Likert scale (1 = “Never” to 5 = “Very often”) regarding their gaming behaviors over the past 12 months. The total score ranges from 9 to 45, with higher scores indicating severe internet gaming disorder issues. To diagnose IGD, participants must have selected at least five items with the “very often” response. The Brazilian version performed well, achieving a total content validity coefficient of 0.96 and receiving positive qualitative assessment from experts in internet gaming disorder.

The Depression, Anxiety, and Stress Scale (DASS-21) (Lovibond & Lovibond, 1995; Vignola & Tucci, 2014) was used to assess symptoms of depression, anxiety, and stress. DASS-21 is a three-dimensional scale that measures these symptoms using 21 items answered on a 4-point Likert scale (from zero, “0”, meaning “It did not apply at all”, to four, “4”, meaning “Applied a lot, or most of the time”). Participants indicate how much they experienced each symptom in the past week. The total score ranges from 0 to 42, with symptom severity classified from “Normal” to “Extremely severe”, whilst higher scores reflect severe symptoms. The scale demonstrates good internal consistency, with coefficients ranging from 0.86 to 0.92 for each subscale. Additionally, there are strong correlations with scales that measure the same constructs.

## Procedures

The translation of OGD-Q followed the steps outlined by Guillemin et al. (1993) and the guidelines of the International Test Commission (2018). These steps included obtaining permission from the scale’s author, conducting two independent translations, creating a consensus version from these translations, submitting the version for review by field experts, performing the back-translation, and sending it for evaluation by the professionals responsible for the original version (Guillemin et al., 1993; International Test Commission, 2018).

This project followed the guidelines established by the National Health Council and the Brazilian Federal Council of Psychology and was approved successfully. Participants only responded after reading and accepting the Informed Consent Form (TCLE). The survey was created using FormR software (R Core Team, 2025) and was promoted online through the laboratory’s social media.

### Data Analysis

For the data analysis, we partly followed the plan from the original article (González-Cabrera et al., 2020). We conducted the analysis using the JASP statistical program (JASP Team, 2025). For descriptive analyses, we assessed the normality of continuous variables using the Shapiro-Wilk test, calculated scores for the OGD-Q, IGDS9-SF, and DASS-21, and determined the prevalence of participants in each category measured by the OGD-Q.

The main analysis conducted was a Confirmatory Factor Analysis (CFA) aimed at evaluating the structure of the instrument. We suggested testing a unifactorial solution based on the original framework developed by González-Cabrera et al. (2020).

The scale items were treated as ordinal variables, considering their Likert-type format. Consequently, the model was estimated using the Diagonally Weighted Least Squares (DWLS) method (Kline, 2016; Li, 2016). This methodology utilizes the polychoric correlation matrix to fit the model. The estimated parameters included factor loadings, residual variances, and average variance extracted (AVE). The factor variance was fixed to a constant for model identification, and thus its significance was not tested. Factor loadings with values ≥ 0.40 are considered acceptable for interpreting item contributions to the latent factor (Kline, 2016). For residual variances, low values are ideal (≤ 0.50) (Kline, 2016). For average variance extracted, values ≥ 0.50 are considered suitable (Hair et al., 2010).

The adequacy indices examined included the chi-square significance test (χ2), the comparative fit index (CFI), the non-normative fit index (NNFI), the Steiger root mean square error of approximation (RMSEA), the root mean squared residual (SRMR), and the comparison fit index (CFI). Fit indices were evaluated according to established thresholds, RMSEA ≤ 0.08, CFI ≥ 0.90, NNFI/TLI ≥ 0.90, and SRMR ≤ 0.08 (Alavi et al., 2020; Hu & Bentler, 1999; Kline, 2016; Xia & Yang, 2019). Regarding the chi-square significance test, aside from a higher p-value, considering its sensitivity to sample size, an x/df ratio less than 5 is a good guideline (Alavi et al., 2020; Kline, 2016).

Because the data were nonparametric, we assessed the scale’s nomothetic validity using Spearman’s Rho correlations with the IGDS9-SF (Donadon et al., 2020) and, separately, with the three dimensions of the DASS-21 (Vignola & Tucci, 2014), following the same procedure as the original study. We assessed internal consistency using Cronbach’s alpha and Omega coefficients. For significant p-values, we considered *p* < 0.05.

## Results

### Demographic Results

The final sample comprised 191 women (64%) and 107 men (36%). Further sociodemographic characteristics of the participants, including age, education, income, and occupation, are detailed in Table 1.

**Table 1:** Sociodemographic characteristics

Variable	*n* (%)
Sex	
Female	191 (64.1)
Male	107 (35.9)
Age (years)	33,0(8,6)¹
Education	
Completed University Education	187 (62,7)
Incompleted University Education/ Currently Enrollled	81 (27,1)
Complete High School	28 (9,3)
Incomplete High School	2 (0,6)
Monthly Income	
Bellow average	34 (11,4)
Average	124 (41,6)
Above average	140 (47,0)
Occupation	
Working Only	157 (52,7)
Working and Studying	63 (21,1)
Studying Only	53 (17,8)
Others	25 (8,4)

### Translation and Adaptation

We obtained approval from the original authors to translate and adapt the OGD-Q into Brazilian Portuguese. The principal author of the OGD-Q agreed to review the back-translated version. We invited two Brazilians fluent in Spanish to translate the questionnaire, each given different instructions. One was asked to produce a literal translation (TL), while the other focused on conveying meaning (TS). The resulting versions are shown in Table 2. Some differences are notable: in item 1, the TL used “conseguir a euforia”, while the TS used “conseguir a aposta mais alta”. In item 7, the TS omitted the phrase “não conseguir parar de jogar quando quer…” used in the TL. In item 11, the TS added “dormir menos que de costume caso jogue à noite,” whereas the TL included “sair menos se joga à noite.” Overall, the TL used “jogo de aposta online”, but also “jogo de azar online” while the TS used “jogo online de aposta”.

To create a unified version (VU), both versions (TL and TS) were analyzed and combined. VU is shown in Table 2. A critical point in translation and adaptation concerns the nomenclature of gambling behavior. The term “jogo de azar” was excluded to prevent confusion. It was formerly more commonly used in Brazil, but “azar” refers to “bad luck” in Brazilian Portuguese, and the growth of gambling practices appears to have led to changes in the term usage. Currently, the term “azar” is avoided by the gambling industry and in assessment instruments because it has an adverse desirability effect on responses. Thus, we used “jogo de aposta online” to refer to online gambling. The term “aposta” was included to avoid ambiguity with “jogo online”, since “jogo” means both gamble and game. Thus, to avoid confusion with online video game activities, the professional linguist suggested this change, which the team accepted. It is worth noting that we considered using only “aposta”, but two main points led us to conclude that retaining “jogo de” was necessary. First, Brazilian official terminology for online gambling condition is “transtorno do jogo” in the two main technical mental disorder manuals (DSM-5 and ICD-11) (American Psychiatric Association, 2022; World Health Organization, 2019). Because instruments are also means of information, the exclusion of remembering the formal terms involved in the condition could help to promote misinformation and lack of literacy about gambling disorder, which is in the opposite direction of our intentions (Valentim, 2024). Second, the ITC guidelines (International Test Commission, 2018) emphasize the importance of cultural and linguistic equivalence in the translation of measures. Thus, while aiming to preserve both literal and conceptual meanings, we retained the three-term structure, replacing only “azar” with “aposta”.

In item 1, we used “conseguir a euforia”. In item 7, we included the phrase “não conseguir parar de jogar quando quer…” which was omitted by the TS. In item 11, we used the TL “sair menos se joga à noite.” After finalizing the unified version, a certified professional translator fluent in Spanish translated VU into Spanish, resulting in a back-translated version (VR) shown in Table 2, which was reviewed by the first author of the OGD-Q. While the original title was “Cuestionario sobre juego patológico online (OGD-Q)”, the title of the VR was “Cuestionario sobre transtornos del juego en línea (OGD-Q)”. In general, the author said that the VR was more formal than the original, which was intended to be more informal: in item 1, changing “te sientes” to “se siente”, and replacing the pronoun “te” with “se” in items 2 and 6, although the author did not request these changes. At the start of the questionnaire, it was requested to change “caja fuerte” to “cofres” when referring to loot boxes. In item 1, the translation of “subidón” was criticized. Item 8 was considered the most different, as the question was asked in the entire sentence (“¿Regresa a menudo al juego para intentar recuperar dinero después de perder en una apuesta o juego de aposta en línea?”), whereas in the original, only the last sentence was used (“Después de perder dinero en una apuesta o en un juego de apuesta online, ¿sueles volver a jugar para intentar recuperar ese dinero?”). Considering the suggestions received, in item 1 we changed the translation of “subidón” to “prazer” instead of “satisfação”. In item 8, we maintained the structure of the original version.

The initial version of the scale, already improved based on the original author’s feedback, was reviewed by five independent experts in psychology. All experts were psychologists with more than 5 years of clinical experience and postgraduate degrees in psychology. They were asked to evaluate the clarity, theoretical relevance, and representativeness of each item and the overall scale using a 4-point Likert scale (1 = Not Relevant, 2 = Slightly Relevant, 3 = Quite Relevant, and 4 = Highly Relevant). All experts reached a unanimous agreement, which we attribute to the previous refinement of the instrument. Because of this consensus, the experts confirmed the scale’s satisfactory content validity.

We revised the final linguistic translation to Brazilian Portuguese and produced the final version of the OGD-Q-BR. The final translated instrument is included in APPENDIX A. The translation process of the OGD-Q instructions and the OGD-Q response scale are available in APPENDIX B and APPENDIX C, respectively.

### Descriptive Analyses

The normality test showed that our sample has a non-normal distribution, as indicated by a significant Shapiro-Wilk p-value (*p* < 0.001) for all variables. The mean for OGD-Q was 12.5 (SD = 4.7), for IGDS9-SF was 11.9 (SD = 5.8), and the total DASS-21 score was 17.9 (SD = 11.7).

Regarding the prevalence of participants at least at risk for the development of gambling disorder, 7 participants have met the criteria. Within the total sample size, participants categorized as having a gambling disorder (meeting 4 or more criteria for 12 months or more) accounted for 0.67% (*n* = 2). Those experiencing problems with online gambling (meeting 4 or more criteria for a period of 6 to 12 months) represented 1% (*n* = 3), while participants at risk (meeting 4 or more criteria for less than 6 months) also made up 0.67% (*n* = 2). The scores for participants in the categories above ranged from 27 to 60, spanning the continuum from at-risk status to gambling disorder. In the original study by Gonzalez Cabrera, which included 883 participants, 0.89% (*n* = 24) were identified as having an online gambling disorder, 0.77% (*n* = 21) reported problems with online gambling, and 0.56% (*n* = 15) were considered at risk of developing issues.

**Table 2:** Phases of translation of the OGD-Q-BR

Item	Original	Unified Translation	Backtranslation	Final Version
**1.**	¿Sientes la necesidad de gastar cada vez más dinero para conseguir el subidón que deseas?	Você sente a necessidade de gastar cada vez mais dinheiro para conseguir a euforia que deseja?	¿Siente la necesidad de gastar cada vez más dinero para conseguir lo placer que desea?	Você sente a necessidade de gastar cada vez mais dinheiro para conseguir o prazer que deseja?
**2.**	¿Te sientes nervioso, irritado o enfadado cuando intentas reducir o dejar el juego de azar online?	Você se sente nervoso, irritado ou chateado quando tenta reduzir ou parar o jogo de aposta online?	¿Se siente nervioso, irritado o aburrido cuando intenta reducir o detener su juego de apuestas en línea?	Você se sente nervoso, irritado ou chateado quando tenta reduzir ou parar o seu jogo de aposta online?
**3.**	¿Has intentado controlar, reducir o abandonar el juego de azar online y no has podido hacerlo? (si no juegas nada, marca la opción nunca)	Você já tentou controlar, reduzir ou abandonar o jogo de aposta online e não conseguiu? (se não joga nada, marque a opção nunca)	¿Alguna vez ha intentado controlar, reducir o abandonar el juego de apuestas en línea y ha fracasado? (si no juega, marque la opción Nunca).	Você já tentou controlar, reduzir ou abandonar o jogo de aposta online e não conseguiu? (caso não jogue, marque a opção nunca).
**4.**	¿Has sentido alguna vez que el juego de azar online ha tenido consecuencias negativas a nivel personal, social, familiar o académico/laboral, y aun así has seguido jugando?	Você sentiu que alguma vez a prática do jogo de aposta online trouxe consequências negativas no âmbito pessoal, social, familiar ou acadêmico/trabalho, mas ainda assim continuou jogando?	¿Alguna vez ha sentido que el juego de apuestas en línea ha tenido consecuencias negativas a nivel personal, social, familiar o académico/laboral, y aún así continuó jugando de todos modos?	Você já sentiu que a prática do jogo de aposta online teve consequências negativas a nível pessoal, social, familiar ou acadêmico/trabalho, e continuou jogando mesmo assim?
**5.**	¿Piensas a menudo en las apuestas online, por ejemplo, recordando apuestas pasadas, planificando tus próximas apuestas, pensando en formas de ganar más dinero jugando online, reviviendo algunos momentos relacionados con el juego de azar online, etc.?	Você pensa frequentemente nas apostas online, por exemplo, lembrando apostas passadas, planejando suas próximas apostas, pensando em formas de ganhar mais dinheiro jogando online, revivendo momentos relacionados ao jogo de aposta online, etc.?	¿Piensa a menudo en las apuestas en línea, por ejemplo, recordando apuestas pasadas, planificando apuestas futuras, pensando en formas de ganar más dinero jugando en línea, reviviendo momentos relacionados con los juegos de apuestas en línea, etc.?	Você pensa frequentemente nas apostas online, por exemplo, lembrando apostas passadas, planejando próximas apostas, pensando em formas de ganhar mais dinheiro jogando online, revivendo momentos relacionados ao jogo de aposta online, etc.?
**6.**	¿Apuestas o juegas a juegos de azar online cuando te encuentras triste, ansioso o te sientes culpable, para sentirte mejor o dejar de pensar en cómo te sientes?	Você aposta ou joga jogos de aposta online quando está triste, ansioso ou se sente culpado, para se sentir melhor ou deixar de pensar em como está se sentindo?	¿Apuesta o juega en línea cuando está triste, ansioso o se siente culpable, para sentirse mejor o dejar de pensar en cómo se siente?	Você aposta ou joga jogos de aposta online quando está triste, ansioso ou se sente culpado, para se sentir melhor ou deixar de pensar em como está se sentindo?
**8.**	¿Sientes que tienes poco control sobre el juego de azar online (p. ej., jugar más de lo que te gustaría, gastar más dinero de lo que quisieras, jugar en sitios en los que no deberías hacer eso, no poder parar de jugar cuando quieres…)?	Você sente que tem pouco controle sobre o jogo de aposta online (por exemplo, jogar mais do que gostaria, gastar mais dinheiro do que queria, jogar em sites onde não deveria fazer isso, não conseguir parar de jogar quando quer…)?	¿Siente que tiene poco control sobre los juegos de apuestas en línea (por ejemplo, juega más de lo que le gustaría, gasta más dinero del que pretendía, juega en lugares donde no debería, no puede dejar de jugar cuando quiere)?	Você sente que tem pouco controle sobre o jogo de aposta online (por exemplo, joga mais do que gostaria, gasta mais dinheiro do que pretendia, joga em lugares onde não deveria fazer isso, não consegue parar de jogar quando quer)?
**9.**	Después de perder dinero en una apuesta o en un juego de azar online, ¿sueles volver a jugar para intentar recuperar ese dinero?	Depois de perder seu dinheiro em uma aposta em um jogo de aposta online, você costuma voltar a jogar para tentar recuperar esse dinheiro?	Después de perder dinero en un juego de apuestas online, ¿vuelves a jugar para intentar recuperar el dinero perdido?	Depois de perder dinheiro em um jogo de apostas online, você volta a jogar para tentar recuperar o dinheiro perdido?
**10.**	¿Mientes a los demás para ocultar cuánto tiempo juegos o cuánto gastas realmente en juegos de aposta online?	Você costuma mentir para as pessoas para esconder o tempo que passa ou quanto dinheiro gasta jogando os jogos de aposta online?	¿Miente a la gente para ocultar cuánto tiempo o dinero realmente gasta jugando juegos de apuestas en línea?	Você mente para pessoas para esconder o tempo que passa ou quanto dinheiro realmente gasta jogando jogos de aposta online?
**11.**	¿Has pedido dinero a alguien para mejorar o superar la mala situación económica que te ha causado el juego de azar online?	Você já pediu dinheiro a alguém para melhorar ou superar a má situação econômica que o jogo de aposta online te causou?	¿Alguna vez le ha pedido dinero a alguien para mejorar o salir de un problema financiero que le causó el juego de apuestas en línea?	Você já pediu dinheiro a alguém para melhorar ou sair de um problema financeiro que o jogo de aposta online te causou?
**12.**	¿Has sentido que dabas prioridad al juego de azar por encima de otras áreas de tu vida que antes habían sido más importantes (p. ej. Estudiar, salir con amigos, dormir menos si juegas de noche, etc.)?	Você sente que dava prioridade ao jogo online de aposta ao invés de outras áreas da sua vida que antes eram mais importantes (estudar, sair com seus amigos, dormir menos que de costume caso jogue a noite, etc.)?	¿Alguna vez ha sentido que prioriza el juego de apuestas en línea sobre otras áreas de su vida que antes eran más importantes (estudiar, salir con sus amigos, dormir menos de lo habitual si juega por la noche, etc.)?	Você já sentiu que dá prioridade ao jogo de aposta online ao invés de outras áreas da sua vida que antes eram mais importantes (estudar, sair com seus amigos, dormir menos que de costume caso jogue a noite, etc.)?

### Factor Structure of the Brazilian Version of OGD-Q

We conducted CFA testing of the one-dimensional model. The one-factor model produced the following results: χ2 = 32.868, df = 44, *p* = 0.891, RMSEA = 0.000 (90% CI [0.000, 0.019]), CFI = 1.000, NNFI = 1.001, SRMR = 0.052. Internal consistency values were 0.92 for Cronbach’s Alpha and 0.93 for Omega. All factor loadings were significant and ranged from 0.66 to 0.9 (see Table 3), exceeding the 0.40 criterion (Kline, 2016). The Average Variance Extracted (AVE) was 0.789, also exceeding the ≥ 0.50 threshold (Hair et al., 2010), confirming that the latent factor explains 78.9% of the item variance. Residual variances ranged from 0.048 to 0.569. While the majority were below the ideal ≤ 0.50 cutoff (Kline, 2016), the overall high AVE indicates that the construct suggests good cohesion despite one item having slightly higher measurement error.

Overall, the model indicates adequate fit indices and parameter estimates, confirming the quality of the one-factor model, consistent with the original study (González-Cabrera et al., 2020).

**Table 3:** Factors loading of the confirmatory factor analysis of the OGD-Q-BR

Factor						95% Confidence Interval		
	Indicator	Estimate	Std. Error	z-value	*p*	Lower	Upper	Std. Est. (all)
OGD-Q	ogd1	0.657	0.060	10.862	< 0.001	0.538	0.775	0.657
	ogd2	0.873	0.039	22.617	< 0.001	0.797	0.949	0.873
	ogd3	0.928	0.036	25.766	< 0.001	0.857	0.999	0.928
	ogd4	0.910	0.033	27.595	< 0.001	0.845	0.975	0.910
	ogd5	0.910	0.027	33.584	< 0.001	0.857	0.963	0.910
	ogd6	0.976	0.015	65.054	< 0.001	0.946	1.005	0.976
	ogd7	0.937	0.031	30.611	< 0.001	0.877	0.997	0.937
	ogd8	0.882	0.033	26.582	< 0.001	0.817	0.947	0.882
	ogd9	0.943	0.026	36.530	< 0.001	0.892	0.993	0.943
	ogd10	0.750	0.036	21.006	< 0.001	0.680	0.820	0.750
	ogd11	0.956	0.022	43.524	< 0.001	0.913	0.999	0.956

### Nomothetic Validity

Following the original article, we performed correlations between OGD-Q and measures of online gaming and depression, anxiety, and stress symptoms. We used the Spearman Rho correlation test due to the non-normality of the sample. The score of the OGD-Q correlated with the scores of the IGDS9-SF and DASS-21, considering the total sample and separately as a function of sex. Considering total sample correlations, OGD-Q correlated with IGDS9-SF (*r* = 0.24, *p* < 0.001), DASS-21 total (*r* = 0.13, *p* < 0.05), DASS-21 stress (*r* = 0.11, *p* < 0.05), DASS-21 anxiety (*r* = 0.19, *p* < 0.001), and no significant correlation was found for DASS-21 depression. Additionally, we performed separate correlations by sex, with correlation values for women shown above the diagonal and values for men below it in Table 4.

**Table 4:** Correlations by sex between OGD-Q-BR- x DASS-21 x IGDS9-SF

Variable		1.	2.	3.	4.	5.	6
1.ODG-Q-BR Score	Spearman´s rho	—	0.298**	0.115	0.060	0.201**	0.067
2. IGDS9-SF Score	Spearman´s rho	0.163	—	0.139	0.154*	0.150*	0.052
3. DASS21 Total Score	Spearman´s rho	0.194*	0.299**	—	0.879**	0.813**	0.869**
4. Stress Score (DASS21)	Spearman´s rho	0.215*	0.324**	0.894**	—	0.649**	0.627**
5. Anxiety Score (DASS21)	Spearman´s rho	0.215*	0.231*	0.685**	0.491**	—	0.572**
6. Depression Score (DASS21)	Spearman´s rho	0.124	0.247**	0.905**	0.704**	0.493**	—

Values for correlations with women only are shown above the diagonal, while those with men only are below the diagonal. ** *p* < 0.001. * *p* < 0.05

## Discussion

This study aimed to translate the Online Gambling Disorder Questionnaire (OGD-Q) into Brazilian Portuguese and to validate and adapt it for use in Brazilian adults. Such effort is needed to enable replication of the measure across cultures, as noted by the original study (González-Cabrera et al., 2020), and to investigate the current health problem of online gambling behavior in Brazil. The OGD-Q (González-Cabrera et al., 2020) is the first instrument designed for assessing the symptoms of gambling disorder, focusing on the online format of gambling, based on the nine criteria of gambling disorder in the DSM-5-TR (American Psychiatric Association, 2022), the ICD-11 (World Health Organization, 2019), and expert recommendations (González-Cabrera et al., 2020). 

The translated version yielded a one-factor model with good fit indices, confirming the hypothesized one-dimensional structure of the instrument and replicating the result reported by González-Cabrera et al. (2020). Additionally, it had high internal consistency coefficients, with Cronbach’s alpha of 0.92 and Omega of 0.93 in the Brazilian sample, consistent with those reported by González-Cabrera et al. (2020), which were 0.94 and 0.95, respectively, as well as those by Stavropoulos et al. (2022), which were both 0.95. Our standardized factor loadings were high, all exceeding 0.68, consistent with González-Cabrera et al. (2020), who reported that all loadings exceeded 0.63. Other parameter estimates, residual variances, and AVE were also within the established thresholds. However, they are not comparable since González-Cabrera (2020) and Stavropoulos et al. (2022) did not evaluate them. Overall, our factor-structure evidence is strongly consistent with that of the original study (González-Cabrera et al., 2020). Regarding the later replication by Stavropoulos et al. (2022), which reported factor loadings for all items ranging from 0.87 to 0.97, most of our items fell within these ranges. However, in Stavropoulos et al. (2022), two items performed better than in our study (items 1 and 10), which may be a simple variation across samples.

Significant positive correlations between OGD-Q and the DASS-21 total, anxiety, and stress subscales were observed across all participants and were interpreted as nomothetic outcomes. These support the idea that online gambling disorder symptoms involve a broader psychological network linked to general distress, anxiety, and stress. Besides revealing this psychometric indicator, the results also support previous research (Gomez et al., 2022), including González-Cabrera et al. (2020) initial work. We also found that OGD-Q was positively correlated with IGDS9-SF in the entire sample. It measures internet gaming disorder symptoms and has previously been positively associated with the OGD-Q (Beranuy et al., 2020; Gomez et al., 2021, 2023; González-Cabrera et al., 2024). Both the IGDS9-SF and OGD-Q are based on shared mechanisms related to addictive disorders, sharing characteristics such as tolerance, relapse, withdrawal, and features of the online environment. Repeatedly, significant positive relationships among these measures were observed (Beranuy et al., 2020; Gomez et al., 2021, 2023; González-Cabrera et al., 2024), providing evidence for the concurrent validity of the Brazilian version of the OGD-Q within a nomothetic framework, given the relevant external similarities between the two phenomena.

Unlike IGDS9-SF, DASS-21 total, anxiety, and stress, OGD-Q did not correlate with DASS-21 depression score for the whole sample. It can be a product of different factors. One, depression and online gambling are not necessarily associated, particularly in a non-clinical sample as ours. Second, the correlation was not entirely null; it was significant, albeit weak, for women, which highlights the need for idiographic assessments. Since the nomothetic approach provides only an initial overview and human functioning requires a deeper understanding, we adopted an idiographic approach to examine sex-specific associations (Kuper et al., 2024). We observed significant correlations for both women and men with OGD-Q and DASS-21 scores, including total, anxiety, and stress. Although significant, these correlations remained low. In fact, these findings were like those reported by González-Cabrera et al. (2020). Such associations were expected to be at most moderate because online gambling and DASS-21 symptoms are related but not identical variables. Additionally, because our study was not conducted with a clinical sample, symptoms were underrepresented, which limited variation. Although further research on OGD-Q in clinical populations is needed, our primary aim was to test it in a community sample to begin outreach to the general population.

Unlike the DASS-21, the IGDS9-SF (internet gaming disorder symptoms) was not consistently significantly associated with the OGD-Q across both sexes; it was significant for women but not for men. This conflicts with existing literature, which shows that internet gaming and online gambling are linked in both women and men (Mallorquí-Bagué et al., 2017; Medeiros et al., 2015; Rozgonjuk et al., 2023), especially in the first publication of the OGD-Q (González-Cabrera et al., 2020). Our unusual results may be due to the small number of men in the sample (*n* = 107). It is also worth noting that, although not universally accepted, some studies suggest that problematic gambling and problematic gaming may not always overlap because they are different constructs, indicating that other underlying variables could influence these relationships (Delfabbro & King, 2020; Sanders & Williams, 2019). Therefore, our conflicting findings with the original work about the link between internet gaming and online gambling may reflect inconsistencies in the OGD-Q, or they could indicate sex-specific differences in the development of symptoms for internet gaming and online gambling disorders, opening perspectives for scientific investigation. Additionally, it is important to mention that in the original study (González-Cabrera et al., 2020), participants were adolescents, whereas we evaluated adults. Since age affects behavioral manifestations, these differences might also be related to age (Kluwe-Schiavon et al., 2016). As with age, cultural and other individual differences between samples could have contributed to the observed variation, which warrants further research to enhance understanding of these differences.

Regarding DASS-21 correlations, the results are consistent with those reported by González-Cabrera et al. (2020), who also found significant correlations for both women and men across all DASS-21 subscales with OGD-Q. Additionally, although the correlations reported by González-Cabrera et al. (2020) were considered significant, they were low for total scores and for scores for both men and women. This aligns with our own weak correlations. However, we did not expect to find high correlation indices, as the constructs are not identical but rather theoretically related variables, and this study aimed to develop an instrument for use in the general population rather than in clinical samples.

Regarding the prevalence rates of participants in each category measured by OGD-Q, we have found low prevalences spanning from individuals at-risk to those with online gambling disorder. Although absolute percentages and sample sizes differ, distribution across categories (gambling disorder, problems with online gambling, and at-risk status) followed a similar pattern of low prevalence, also found in the original study (González-Cabrera et al., 2020). In addition, given the low prevalence observed, it was not possible to conduct analyses to compare gambling profiles or to determine them. This low prevalence is nevertheless consistent with the original study, although the exact percentages differ.

When discussing the translation process, several important points must be highlighted. In Brazil, the term for gambling disorder is “Transtorno do Jogo.” However, “jogo” in Brazilian Portuguese is ambiguous, as it can refer to both video games (“jogos de video game”) and gambling activities (“jogos de azar” or “aposta”). Additionally, there is no direct equivalent in Portuguese for the word “gambling,” which encompasses all forms of gambling activities. In Brazil, we have “jogos de azar” or “aposta,” but these terms do not fully capture the same meaning. The term “jogos de azar” (games of bad luck) is particularly problematic, as it carries a negative connotation and is not commonly used in Brazilian society, where gambling activities are typically viewed as a form of entertainment. Given these factors, our translation process, guided by expert evaluations, led us to adopt the term “jogos de aposta online” for online gambling activities in Brazil. Although we conducted a careful translation process with expert feedback, we recognize that this terminology has limitations and requires further qualitative validation, particularly through cognitive interviewing.

In addition to terminology, theoretical aspects require attention. The OGD-Q instructions lists different gambling methods (e.g., bingo, poker, etc.), including “loot boxes”. There are discussions whether loot boxes are a form of gambling or if it involves gambling-like mechanisms, without consensus in the literature and implicating in a variety of legal determinations among different countries (Griffiths, 2018; Drummond & Sauer, 2018; Xiao et al., 2022). González-Cabrera et al. (2020) recognized that loot boxes are not legally considered online gambling; however, they emphasized the overlap it has with psychological characteristics of gambling. To avoid discharacterization of the measure and following González-Cabrera et al. (2020) assumptions we kept it in the translated instrument. And also, since young people who play video games are already familiar with this terminology, referring to loot boxes can help reach a wider audience. By characterizing loot boxes in the instructions, the aim is to clarify the concept rather than complicate it, and this approach is not expected to introduce bias into the construct measured. However, OGD-Q aims to screen general symptoms of online gambling in the general population, rather than gambling-like digital behaviors. Therefore, interpretation of the scores should be considered in both clinical and epidemiological areas. Thus, if OGD-Q is used clinically, further investigations are required to interpretate how online gambling manifests in each person. High scores of individuals who primarily purchase in loot boxes but do not engage in traditional monetary gambling should be interpreted with caution. However, research shows the relationship between loot boxes and problem gambling, for that reason the results should be closely putten together with clinical observation and taking into consideration the law of the country about whether loot boxes are a gambling format or not (Drummond & Sauer, 2018; Montiel et al., 2022; Spicer et al., 2022; Zendle et al., 2019). Moreover, OGD-Q does not provide epidemiological documentation on the types and forms of online gambling, which has implications for further studies. We recommend adding questions about gambling behavior to understand which gambling formats are most common. This way, it is possible to distinguish between individuals who primarily engage in loot boxes, bingo sports bets, or other behaviors. It is important to stratify prevalence estimates by type of gambling behavior to avoid inflating rates with individuals who engage in loot boxes but do not participate in traditional real-money gambling formats.

The procedures and results of our work ensure safety and psychometric quality for using the Portuguese Brazilian version of OGD-Q; however, the study has some limitations that should be considered. Firstly, the sample size was small and predominantly composed of women, which means it does not equally represent both sexes. Assuming that gambling is more common among men, a detailed investigation in a future sample could improve psychometric evidence for the measure. Secondly, the sample did not largely include individuals with gambling disorder because a clinical sample was not the focus. Future studies with larger and heterogeneous samples should investigate measurement invariance across demographic groups, such as sex, age, and education, to ensure the instrument functions consistently. This can be achieved by conducting multi-group factor analysis to assess equivalence between these groups. They should also test validity and measurement invariance by comparing clinical and community samples. Future research should explore the psychometric properties of this instrument in other Brazilian samples, particularly among problematic and pathological gamblers. Additionally, the psychometric rigor would be enhanced by conducting a test-retest reliability assessment and utilizing longitudinal studies to assess the instrument’s predictive validity.

Despite these limitations, the study has a significant strength: it offers valuable practical and research implications for the field of gambling disorder research and assessment in Brazil. Notably, it is the first psychometric instrument specifically designed to evaluate gambling disorder in the online domain in Brazil.

## Conclusion

Based on the evidence of this study, we conclude that the Online Gambling Disorder Questionnaire (OGD-Q) Brazilian Version is adequately capable of assessing online gambling disorder symptoms in the general Brazilian population. Therefore, the instrument and its correction are available in the appendix below.

## Electronic Supplementary Material

Below is the link to the electronic supplementary material.

Supplementary Material 1 (DOCX 185 mb)

## Data Availability

The datasets generated during and/or analyzed during the current study are available from the corresponding author on reasonable request.

## Appendix A

**Questionário sobre Transtorno do Jogo de Aposta Online – Versão Brasileira**.

**No questionário a seguir você vai encontrar afirmações relacionadas ao jogo de aposta online. Isso se refere a todos os jogos online que envolvem a sorte**,** por exemplo**,** partidas de pôquer online**,** bingos online**,** casas de apostas online**,** além da compra de pacotes**,** caixas de itens aleatórios (as loot boxes) em videogames (FIFA**,** Hearthstone: Heroes of Warcraft**,** CS-GO**,** etc.). Podemos chamar de jogo de aposta online qualquer aposta através da internet**,** com dinheiro**,** na qual você não sabe se ganhará o prêmio desejado. Com isto em mente**,** responda as perguntas a seguir**,** por favor**:

**1 - Nunca/2- Poucas vezes/3 - Com frequência/4 - Com muita frequência/5- Todos os dias**.

Você sente a necessidade de gastar cada vez mais dinheiro para conseguir o prazer que deseja?

1. Você sente a necessidade de gastar cada vez mais dinheiro para conseguir o prazer que deseja?
2. Você se sente nervoso, irritado ou chateado quando tenta reduzir ou parar o seu jogo de aposta online?
3. Você já tentou controlar, reduzir ou abandonar o jogo de aposta online e não conseguiu? (caso não jogue, marque a opção nunca).
4. Você já sentiu que a prática do jogo de aposta online teve consequências negativas a nível pessoal, social, familiar ou acadêmico/trabalho, e continuou jogando mesmo assim?
5. Você pensa frequentemente nas apostas online, por exemplo, lembrando apostas passadas, planejando próximas apostas, pensando em formas de ganhar mais dinheiro jogando online, revivendo momentos relacionados ao jogo de aposta online, etc.?
6. Você aposta ou joga jogos de aposta online quando está triste, ansioso ou se sente culpado, para se sentir melhor ou deixar de pensar em como está se sentindo?
7. Você sente que tem pouco controle sobre o jogo de aposta online (por exemplo, joga mais do que gostaria, gasta mais dinheiro do que pretendia, joga em lugares onde não deveria fazer isso, não consegue parar de jogar quando quer)?
8. Depois de perder dinheiro em um jogo de apostas online, você volta a jogar para tentar recuperar o dinheiro perdido?
9. Você mente para pessoas para esconder o tempo que passa ou quanto dinheiro realmente gasta jogando jogos online de aposta?
10. Você já pediu dinheiro a alguém para melhorar ou sair de um problema financeiro que o jogo de aposta online te causou?
11. Você já sentiu que dá prioridade ao jogo de aposta online ao invés de outras áreas da sua vida que antes eram mais importantes (estudar, sair com seus amigos, dormir menos que de costume caso jogue a noite, etc.)?

Se você está vendo essa pergunta, é porque você apresentou em alguma ocasião (com mais ou menos frequência) alguma das situações descritas anteriormente (como sentir vontade de gastar mais dinheiro, se sentir mal por não jogar, sentir que jogar traz consequências negativas, pensar com frequência em apostas online, sentir que tem dificuldade em controlar a vontade de jogar, continuar apostando mesmo que tenha perdido dinheiro, etc.).

12. Desde quando você sente ou passa por isso?

Há mais de 12 meses.

Há mais de 6 meses.

Há mais de 1 mês.

Recentemente.

## Appendix B

**Table:**

Original Version		Back-Translated Version		Unified Version		Final Version
En el siguiente cuestionario encontrarás afirmaciones relacionadas con el juego de azar online. Esto hace referencia a todos los juegos online en los que participa la suerte, por ejemplo, partidas de póker online, bingos online, casas de apuestas online e incluso la compra de sobres, cajas o cofres en videojuegos (FIFA, Hearthstone: Heroes of Warcraft, CS-GO, etc.).Cualquier gasto de dinero para comprar una apuesta donde no sabes si te tocará o no el premio deseado puedes considerarlo un juego de azar online. Teniendo eso en mente, responde a estas preguntas, por favor:		En el siguiente cuestionario encontrará afirmaciones relacionadas con los juegos de azar en línea. Se refiere a todos los juegos en línea que implican suerte, por ejemplo, partidas de póquer en línea, bingo en línea, casas de apuestas en línea, así como la compra de paquetes, cajas o cofres de items aleatorios (loot boxes) en videojuegos (FIFA, Hearthstone: Heroes of Warcraft, CS-GO, etc.).Podemos llamar juego de azar en línea a cualquier apuesta realizada a través de internet, con dinero, en la que no sabe si ganará el premio deseado. Con esto en mente, por favor responda las siguientes preguntas:		No questionário a seguir você vai encontrar afirmações relacionadas ao jogo de azar online. Isso se refere a todos os jogos online que envolvem a sorte, por exemplo, partidas de pôquer online, bingos online, casas de apostas online, além da compra de pacotes, caixas de itens aleatórios (as loot boxes) em videogames (FIFA, Hearthstone: Heroes of Warcraft, CS-GO, etc.).Podemos chamar de jogo de azar online qualquer aposta através da internet, com dinheiro, na qual você não sabe se ganhará o prêmio desejado. Com isto em mente, responda as perguntas a seguir, por favor:		No questionário a seguir você vai encontrar afirmações relacionadas ao jogo de aposta online. Isso se refere a todos os jogos online que envolvem a sorte, por exemplo, partidas de pôquer online, bingos online, casas de apostas online, além da compra de pacotes, caixas de itens aleatórios (as loot boxes) em videogames (FIFA, Hearthstone: Heroes of Warcraft, CS-GO, etc.).Podemos chamar de jogo de aposta online qualquer aposta através da internet, com dinheiro, na qual você não sabe se ganhará o prêmio desejado. Com isto em mente, responda as perguntas a seguir, por favor:

## Appendix C

**Table:**

Original Version	Back-Translated Version	Unified Version	Final Version
1 - Nunca 2 - En alguna ocasión puntual 3 - Con frecuencia 4 - Muy frecuentemente 5 - Todos los días	1 - Nunca 2 - Rara vez 3 - A menudo 4 - Muy a menudo 5 - Todos los días	1 - Nunca 2 - Poucas vezes 3 - Com frequência 4 - Com muita frequência 5 - Todos os dias	1 - Nunca 2 - Poucas vezes 3 - Com frequência 4 - Com muita frequência 5 - Todos os dias
