# Supplementary material for: Translation and Adaptation of the Online Gambling Disorder Questionnaire (OGD-Q) into Brazilian Portuguese
Source: J Gambl Stud. 2026 Mar 8;42(2):531–54. doi: 10.1007/s10899-026-10480-9 (PMC13269456; doi:10.1007/s10899-026-10480-9)
Supplement: Supplementary file 1 — Supplementary Material 1 (DOCX 185 mb) [file 10899_2026_10480_MOESM1_ESM.docx]

**Supplementary File**

**Aminolytic Upcycling of Polyethylene Terephthalate Waste into Functional Aromatic Amide Dye: Synthesis, Structural Characterization, and Application Performance**

Neha Mittal^a^, Rakesh Kumar Soni^a*^

^a^Department of Chemistry, Chaudhary Charan Singh University, Meerut 250004, India

E-mail: [mittaln671@gmail.com](mailto:mittaln671@gmail.com), (orcid.org/0009-0005-2527-4096)

[rksoni_rks@yahoo.com](mailto:rksoni_rks@yahoo.com) (orcid.org/0000-0001-6349-5221)


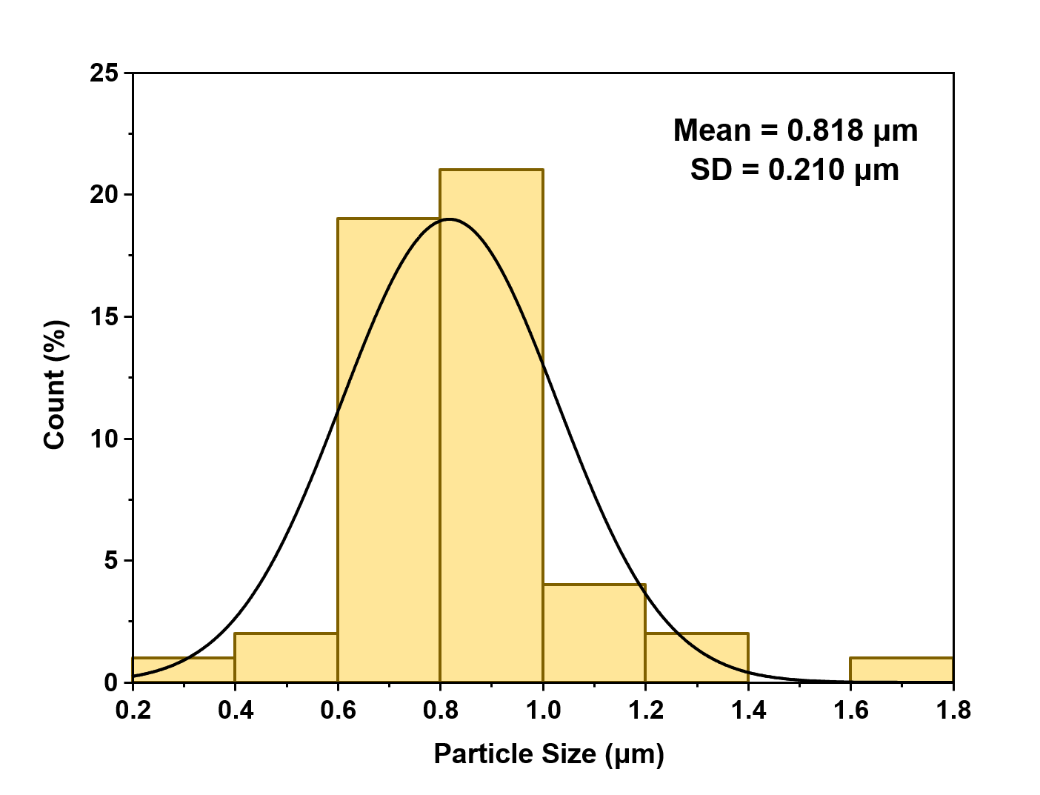


***Figure S1:*** *Particle size distribution histogram of the synthesized dye obtained from ImageJ analysis (n = 50)*

***Table S1:*** *Comparison of observed FTIR absorption bands of the synthesized dye with expected literature ranges and corresponding vibrational assignments*

| **Type of vibration** | **Expected peak range (cm^-1^)** | **Observed peak (cm^-1^)** |
| --- | --- | --- |
| Primary amine group (-NH_2_) stretching | 3200–3400 (often two bands) | 3325, 3211 |
| Aromatic C-H stretching | 3000–3100 | 3044 |
| C=O stretch of amide | 1600–1760 | 1603 |
| C=C stretch (in highly conjugated system) | 1500–1600 | 1507 |
| C-N stretch | 1250–1390 | 1367 |
| C-O stretch | 1000–1300 | 1260 |
| para-substitution | 800–860 | 823 |
| C-H in plane bending | 1000–1300 | 1128 |

***Table S2:*** *Comparison of observed ^1^H NMR chemical shifts of the synthesized dye with expected literature ranges and corresponding proton assignments (DMSO-d_6_)*

| **Type of Proton** | **Expected δ range (ppm)** | **Observed δ (ppm)** |
| --- | --- | --- |
| Ar-**H**_a_ | 6.5 – 8.5 | 7.42 |
| CO-N**H**_b_ | 9.5 – 10.5 | 10.01 |
| Ar-**H**_c_ | 6.5 – 8.5 | 7.06 |
| Ar-**H**_d_ | 6.5 – 8.5 | 6.62 |
| N**H**_e2_ | 3.5 – 5.0 (broad) | 3.65 |

***Table S3:*** *Comparison of observed ^13^C NMR chemical shifts of the synthesized dye with expected literature ranges and corresponding carbon assignments (DMSO-d_6_)*

| **Type of Carbon** | **Expected δ range (ppm)** | **Observed δ (ppm)** |
| --- | --- | --- |
| (Ar)**C**_a_ | 110 – 140 | 137.87 |
| (Ar)**C**_b_-CO | 140 – 160 | 145.89 |
| **C**_c_=O | 160 – 180 | 163.42 |
| (Ar)**C**_d_-NH | 110 – 140 | 122.43 |
| (Ar)**C**_e_ | 110 – 140 | 128.46 |
| (Ar)**C**_f_ | 110 – 140 | 127.16 |
| (Ar)**C**_g_-NH_2_ | 110 – 140 | 114.83 |

***Table S4:*** *Comparison of colorimetric properties and dyeing performance (K/S, L, a*, b*, exhaustion, fixation and fastness ratings) of the present PET-derived aromatic amide dye with commercial blue disperse dyes and other PET-derived disperse dyes*

| **Dye sample** | **Fabric** | **K/S** | **L*** | **a*** | **b*** | **Dye-bath exhaustion (%)** | **Fixation (%)** | **Fastness performance** | **Reference** |
| --- | --- | --- | --- | --- | --- | --- | --- | --- | --- |
| PET-derived aromatic amide dye **(this work)** | Cotton | 12.64 | 28.12 | +1.84 | −14.10 | 85.58% | 96.34% | Excellent heat fastness, excellent to very good wash fastness and good crockfastness | This work |
|  | Polyester | 6.08 | 38.02 | −0.69 | −4.46 | 78.36% | 94.67% |  |  |
| Disperse Blue 79 (Commercial blue disperse dye) | Polyester | 25.89 | 18.38 | 1.82 | -18.51 | 87.52% | — | Good wash fastness, good to moderate light fastness and excellent sublimation fastness | [1] |
|  | Poly(Lactic Acid) Fibre | 31.27 | 20.54 | 9.23 | -26.17 | 58.52% | — |  |  |
| Levafix CA Blue (Commercial blue disperse dye) | polyester/ cotton blend T65/C35 | 4.37 | 49.32 | -4.38 | -30.07 | — | — | Very good crockfastness, excellent to very good wash fastness and good to moderate light fastness | [2] |
|  | polyester/ cotton blend T40/C60 | 7.21 | 50.38 | -4.29 | -28.65 | — | — |  |  |
| PET-derived disperse dye | Polyester | 10.11 | 66.50 | 34.18 | 68.28 | — | — | Good wash fastness and good to moderate light fastness | [3] |
| PET-derived disperse dye | Polyester | 1.99 | 43.03 | 21.31 | 31.05 | — | — |  |  |
| PET-derived bis-azo disperse dye | Polyester | 8.6 | 67.3 | 26.1 | 66.0 | — | — | Very good to excellent wash and sublimation fastness | [4] |
|  | Nylon | 8.7 | 66.8 | 29.9 | 65.5 | — | — |  |  |
| PET-derived bis-azo disperse dye | Polyester | 14.9 | 65.4 | 35.4 | 63.8 | — | — |  |  |
|  | Nylon | 14.9 | 63.4 | 34.7 | 71.0 | — | — |  |  |
| PET-derived bis-azo disperse dye | Polyester | 2.5 | 54.4 | 24.1 | 48.8 | — | — |  |  |
|  | Nylon | 25.5 | 50.5 | 42.2 | 59.5 | — | — |  |  |
| PET-derived bis-azo disperse dye | Polyester | 4.0 | 59.7 | 27.1 | 53.5 | — | — |  |  |
|  | Nylon | 21.5 | 63.2 | 38.9 | 77.7 | — | — |  |  |
| PET-derived bis-azo disperse dye | Polyester | 8.1 | 65.1 | 30.9 | 60.7 | — | — |  |  |
|  | Nylon | 8.1 | 65.1 | 30.9 | 60.7 | — | — |  |  |
| PET-derived bis-azo disperse dye | Polyester | 11.5 | 66.7 | 21.8 | 69.4 | — | — |  |  |
|  | Nylon | 11.5 | 66.7 | 21.8 | 69.4 | — | — |  |  |

**References**

1. Lykidou SS, Pachygiannaki L, Tzouratzoglou E, et al (2025) Performance Evaluation and Fastness Properties of Poly(Lactic Acid) Fibres Dyed with Commercial Disperse Dyes in Comparison to Polyester Fabric. Textiles 5:. https://doi.org/10.3390/textiles5030039

2. Wang Y, Lee CH, Tang AYL, Kan C (2025) Effect of Different Energy Level Disperse Dyes in Dyeability of Polyester/Cotton Blend Fabrics Using PEG-Based Reverse Micelle as Disperse/Reactive Dye Carrier. Fibers Polym 26:2993–3009. https://doi.org/10.1007/s12221-025-00992-3

3. Shukla SR, Harad AM, Jawale LS (2009) Chemical recycling of PET waste into hydrophobic textile dyestuffs. Polymer Degradation and Stability 94:604–609. https://doi.org/10.1016/j.polymdegradstab.2009.01.007

4. Palekar VS, Pingale ND, Shukla SR (2010) Synthesis, spectral properties and application of novel disazo disperse dyes derived from polyester waste. Coloration Technology 126:86–91. https://doi.org/10.1111/j.1478-4408.2010.00231.x
